# Sirtuins and Hypoxia in EMT Control

**DOI:** 10.3390/ph15060737

**Published:** 2022-06-10

**Authors:** Michele Aventaggiato, Federica Barreca, Luigi Sansone, Laura Pellegrini, Matteo A. Russo, Marco Cordani, Marco Tafani

**Affiliations:** 1 Department of Experimental Medicine, Sapienza University, 00161 Rome, Italy; michele.aventaggiato@uniroma1.it (M.A.); federica.barreca@uniroma1.it (F.B.); laura_pellegrini@hotmail.it (L.P.); 2 Laboratory of Cellular and Molecular Pathology, IRCCS San Raffaele Pisana, 00166 Rome, Italy; luigi.sansone@sanraffaele.it (L.S.); matteoantoniorusso44@gmail.com (M.A.R.); 3 MEBIC Consortium, San Raffaele Open University, 00166 Rome, Italy; 4 Department of Biochemistry and Molecular Biology, School of Biology, Complutense University, 28040 Madrid, Spain; marco.cordani@salud.madrid.org; 5 Instituto de Investigaciones Sanitarias San Carlos (IdISSC), 28040 Madrid, Spain

**Keywords:** epithelial–mesenchymal transition, sirtuins, hypoxia, fibrosis, HIF, nanoparticles

## Abstract

Epithelial–mesenchymal transition (EMT), a physiological process during embryogenesis, can become pathological in the presence of different driving forces. Reduced oxygen tension or hypoxia is one of these forces, triggering a large number of molecular pathways with aberrant EMT induction, resulting in cancer and fibrosis onset. Both hypoxia-induced factors, HIF-1α and HIF-2α, act as master transcription factors implicated in EMT. On the other hand, hypoxia-dependent HIF-independent EMT has also been described. Recently, a new class of seven proteins with deacylase activity, called sirtuins, have been implicated in the control of both hypoxia responses, HIF-1α and HIF-2α activation, as well as EMT induction. Intriguingly, different sirtuins have different effects on hypoxia and EMT, acting as either activators or inhibitors, depending on the tissue and cell type. Interestingly, sirtuins and HIF can be activated or inhibited with natural or synthetic molecules. Moreover, recent studies have shown that these natural or synthetic molecules can be better conveyed using nanoparticles, representing a valid strategy for EMT modulation. The following review, by detailing the aspects listed above, summarizes the interplay between hypoxia, sirtuins, and EMT, as well as the possible strategies to modulate them by using a nanoparticle-based approach.

## 1. EMT

### 1.1. Introduction

**Figure 1:**
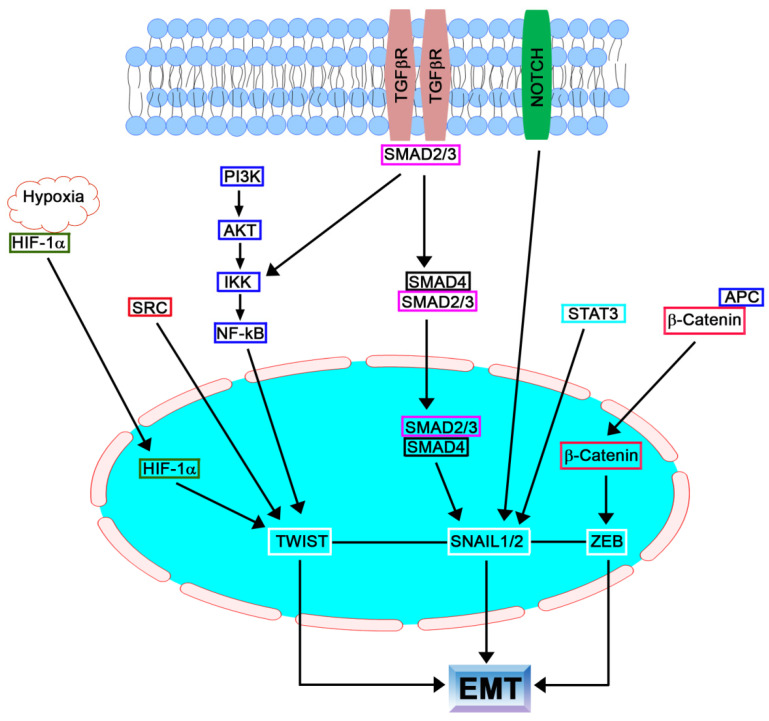
Schematic representation of the different actors and pathways leading to EMT induction.

In the early 1980s, Elizabeth Hay described epithelial to mesenchymal phenotype changes in the primitive streak of chick embryos. She described this process as “epithelial to mesenchymal transformation”. This differentiation process is now commonly known as epithelial–mesenchymal transition (EMT) to emphasize its transient nature, whereas mesenchymal–epithelial transition (MET) describes the reverse process [1,2]. Cells undergoing EMT have typical morphological changes, including the disruption of intercellular junctions, loss of polarity, reorganization of the cytoskeleton, and increased motility [3,4,5,6,7]. The molecular indicators that have been examined to confirm this transformation are: the loss of expression or function of E-cadherin, the reduced abundance of tight-junction proteins zona occludens 1 (ZO-1) and occluding, and the concomitant increase in the mesenchymal markers vimentin, fibronectin, fibroblast-specific protein 1 (FSP-1), α-smooth muscle actin (α-SMA), and N-cadherin [7,8]. The repression of the epithelial phenotype and the activation of the mesenchymal phenotype are due to changes in the expression of some transcription factors, such as SNAIL, TWIST, and zinc-finger E-box-binding (ZEB) [9,10,11,12]. The contribution and expression profile of these transcription factors depends on the cell type and tissue in which the ETM takes place. To complicate the issue further, these same transcription factors can cooperate or antagonize each other during EMT [13,14]. A scheme summarizing the different players and pathways leading to EMT is represented in Figure 1. 

One important aspect to keep in mind when reading this review is that EMT is not a static but a dynamic process and that, when we analyze a tissue or a group of cells in which EMT takes place, we may observe the expression of both epithelial and mesenchymal markers, as well as different morphological features belonging to both epithelial or mesenchymal cells. In fact, epithelial mesenchymal plasticity (EMP) and the hybrid E/M state are new concepts that are adopted to better explain the cellular transition observed in different pathological situations and that result from the interplay between cells and their microenvironment. This has been the subject of two recent reviews by Bornes [15] and Brabletz [16].

### 1.2. SNAIL Family

The proteins of the SNAIL family, SNAIL1 and SNAIL2, activate EMT during development, fibrosis, and cancer [17,18]. SNAIL1 represses E-cadherin expression and recruits the Polycomb repressive complex 2 (PRC2), composed by the methyltransferase enhancer of zeste homologue 2 (EZH2), G9a, the suppressor of variegation 3–9 homologue 1 (SUV39H1), the co-repressor SIN3A, histone deacetylases 1, 2, and/or 3, and the Lys-specific demethylase 1 (LSD1). All these components coordinate the methylation and acetylation of histone H3 Lys 4 (H3K4), H3K9, and H3K27 [19,20]. In addition to repressing epithelial genes, SNAIL1 activates the genes that contribute to the mesenchymal phenotype. This may also involve bivalent domains with, for example, repressive H3K9 trimethylation and the activation of H3K18 acetylation, which enables mesodermal goosecoid (Gsc) expression in response to TGFβ-related signals [21]. The TGFβ- and WNT-family proteins, Notch, and growth factors that act through RTKs all activate SNAIL1 expression, depending on the physiological context. 

### 1.3. TGFβ Family

Transforming growth factor β (TGFβ) is a secreted cytokine that regulates cell proliferation, migration, and the differentiation of a plethora of different cell types. TGFβ elicits a broad range of context-dependent cellular responses and, consequently, alterations in TGFβ signaling have been implicated in many diseases, including cancer [22,23]. The TGFβ ligand activates Smad-dependent and -independent pathways by binding to TGFβ receptors. In Smad-dependent pathways, the activated TGFβ receptor phosphorylates Smad2/3 and accelerates its association with Smad4 and nuclear translocation. The Smad2/3-4 complex promotes the expression of EMT-inducing transcription factors. In the Smad-independent pathway, mitogen-activated protein kinase (MAPK) and phosphatidylinositol 3-kinase (PI3K)/AKT pathways are activated and are required for TGFβ-induced EMT [24,25,26]. The TGFβ family comprises three TGFβs, two activins, many bone morphogenetic proteins (BMPs), and other homodimers and heterodimers of ligands. TGFβ1 and TGFβ2 expression is associated, during development, with EMT-like events, leading to the formation of endocardiac cushions [27], while TGFβ3 is involved in the fusion of the palate [28]. Postnatally, TGFβ1 induces EMT in wound healing, fibrosis, and cancer.

### 1.4. TWIST Family

The TWIST family includes basic helix–loop–helix (bHLH) homodimeric and heterodimeric transcription factors, which function as the main regulators of lineage specification and differentiation [14]. Twist1 contributes to the EMT process and performs essential roles in metastasis and fibrosis [29]. As with SNAIL, TWIST expression downregulates epithelial gene expression and activates mesenchymal gene expression [18]. Twist1 expression is regulated by a large group of upstream regulators or signaling pathways, such as Akt, STAT3, HIF, NF-kB, SRC, the Wnt/β-catenin axis, and TGFβ [30]. Twist induces the loss of E-cadherin-mediated cell–cell adhesion and EMT in epithelial cells. In the case of human invasive lobular carcinomas, an inverse correlation is often visible between the levels of Twist and E-cadherin expression [31].

### 1.5. ZEB Family

ZEB1 and ZEB2, belonging to the ZEB family, can repress or activate transcription by binding regulatory gene sequences to E-boxes [9]. ZEB-mediated transcriptional repression often involves the recruitment of a C-terminal-binding protein (CTBP) co-repressor, although in some cancer cells, ZEB1 represses E-cadherin expression independently of CTBP by recruiting the Switch/sucrose non-fermentable (SWI/SNF) chromatin remodeling protein, BRG1 [32]. When ZEB1 interacts with the transcriptional coactivators, p300/CBP-associated factor (PCAF; also known as KAT2B) and p300, it becomes a transcriptional activator rather than a repressor [33]. Additionally, ZEB1 can recruit the Lys-specific demethylase 1 (LSD1), possibly linking it to histone demethylation in EMT [34]. Therefore, as with SNAIL and TWIST, ZEBs bind E-boxes and function as transcriptional repressors and activators, thereby repressing some epithelial-junction and polarity genes and activating the mesenchymal genes that define the EMT phenotype [13,17,35,36,37]. 

### 1.6. Different Types of EMT

**Figure 2:**
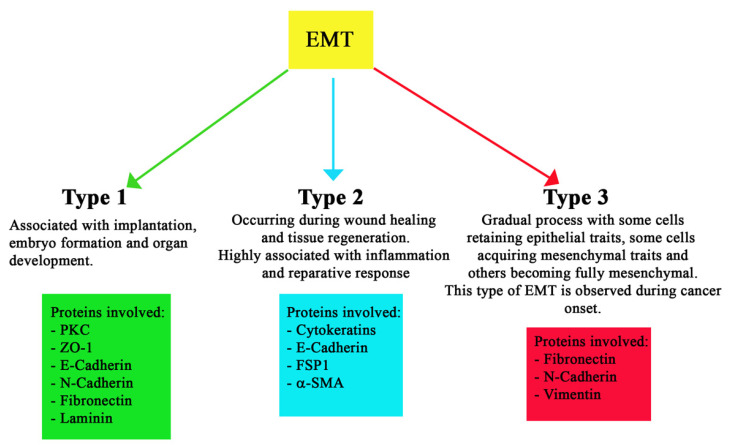
Schematic illustration of the types of EMT. EMT can be differentiated into three types, based on the biological process with which it is associated. Type 1 is mostly associated with development and is characterized by markers such as PKC, ZO-1, etc. [7,36,37,38]. Type 2 is associated with the inflammatory-reparative process, including wound healing, and mostly involves cytoskeletal markers, such as Cytokeratins [39,40]. Type 3 is a gradual process associated with the acquisition of mesenchymal traits in epithelial cells and is mostly involved in cancer development. Its markers are Fibronectin, Vimentin, etc. [41,42,43,44].

Classic or normal EMT is divided into three different types, based on the biological process in which they are involved. Type 1 EMT is typical of embryogenesis, as it facilitates implantation and gastrulation [38]. Wound healing, tissue regeneration, and organ fibrosis are referred to as type 2 EMT. On the other hand, type 3 EMT is associated with tumorigenesis and develops mainly in tumor epithelial cells. This third type helps tumor invasion and metastasis and indicates a worse prognosis. This distinction between the three types of EMT is schematized in Figure 2, which also reports the major proteins involved. 

Type 1 EMT is associated with implantation, embryo formation, and organ development and can generate mesenchymal cells (primary mesenchyme) that have the potential to subsequently undergo a MET to generate secondary epithelia [7]. This type of EMT plays a vital role during gastrulation, when cells organize themselves in the anterior and posterior region of the mesoderm [39]. In type 1, the main expressed markers are: PKC (apical), ZO-1 (tight junction), E-cadherin, N-cadherin (adherens junction), fibronectin, and laminin (BM). Both PKC and ZO-1 are expressed in all epiblast cells, but they are undetectable in mesodermcells. E-cadherin is expressed in epiblast and nascent mesoderm cells, with a gradual shift to N-cadherin in more lateral mesodermcells [40].

Type 2 EMT is activated during wound healing and tissue regeneration in order to reconstruct tissues following trauma and inflammation [41]. The cells involved in type 2 EMT show an epithelial-specific morphology, expressing molecular markers, such as cytokeratin and E-cadherin, together with the ferroptosis suppressor protein 1 (FSP1, an S100 class of cytoskeletal protein) mesenchymal marker and alpha smooth muscle actin (α-SMA) [42].

Type 3 EMT is a gradual process, with some cells retaining epithelial traits, others acquiring mesenchymal traits, and some becoming fully mesenchymal [43]. This type of EMT closely resembles what is now known as hybrid E/M and EMP; in fact, it has been observed in malignant cells acquiring the ability to invade locally and systemically, leading to metastasis, neoangiogenesis, stemness, and therapy resistance [15,16,44,45,46]. In particular, neoangiogenesis has been linked to the overexpression, in tumor cells, of COX2. Recent studies have shown that COX2 participates in cancer invasion and metastasis by decreasing the expression of E-cadherin and leading to phenotypic changes in endothelial and epithelial cells (EMT), increasing neo-vascularization [47]. It is interesting to observe that COX2 expression is driven by NF-kB linking inflammation with tumorigenesis and neovascularization [47]. Moreover, endothelial JAG1, released by tumor cells, can also enhance tumor neoangiogenesis, increasing tumor vessel density and branching by modulating VEGFR2 expression [48].

## 2. EMT in Fibrosis and Cancer

### 2.1. Fibrosis

**Figure 3:**
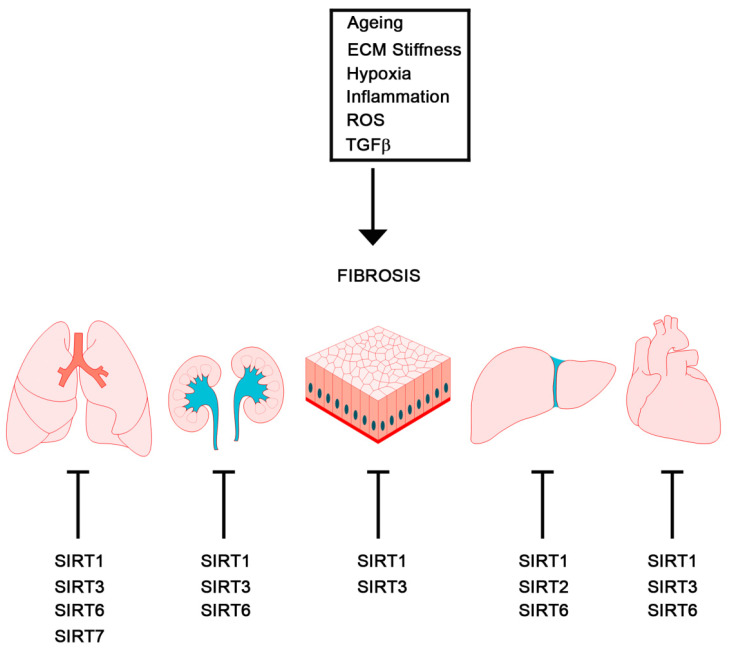
Schematic illustration of different inductors of fibrosis in different organs. For each organ, we have indicated the sirtuins with a known protective effect.

Fibrosis is characterized by an excessive, dysregulated production of extracellular matrix (ECM) proteins, such as type I collagen and fibronectin, by myofibroblasts [49]. The increase in the extracellular matrix leads to the formation of scar tissue and to the loss of the function of the affected organ (such as the skin, kidneys, lungs, cardiovascular system, liver, pancreas, and intestines). Myofibroblasts are the main cell types involved in ECM deposition, which leads to fibrosis [50,51,52,53]. In this context, TGFβ, a profibrotic factor, has an important role in the induction of EMT [54,55,56,57,58,59]. Importantly, recent studies have shown a role of hypoxia in EMT, fibrosis, and cancer, suggesting that hypoxia may play a central role in regulating, as well as being regulated by these events. In fact, while prolonged hypoxia can cause myofibroblast differentiation and fibrogenesis, the presence of fibrosis can reduce oxygen diffusion, creating a hypoxic microenvironment favoring cellular transformation and cancer [55,56,60]. Similarly, the accumulation of myofibroblasts and the alteration in stroma stiffness are recognized as signs of tumor progression and aggressiveness [61]. In this context, Sirtuins, described below, may play an important role. In fact, this family of protein deacylases regulates not only EMT and hypoxic response, as detailed in the sections below, but also fibrosis. In particular, sirtuins mainly regulate fibrosis by inhibiting TGFβ, β-catenin, and by reducing ROS accumulation in different tissues and organs. Figure 3 summarizes the different inductors and players directly or indirectly involved in the fibrosis of different organs, as well as the role of sirtuins.

RENAL FIBROSIS: Interstitial fibrosis characterizes progressive chronic kidney disease, which can lead to tubular atrophy, loss of kidney function and end-stage renal failure [62]. Numerous studies have provided evidence that EMT-derived myofibroblasts originating from tubular epithelia contribute to renal fibrosis and that TGFβ1 is the main inducer of EMT in renal tubular epithelial cells [63]. Importantly, BMP-7 can reverse TGFβ1-induced EMT in tubular epithelial cells by inducing SMAD-dependent E-cadherin expression in vitro. In fact, the systemic administration of recombinant human BMP-7 repaired kidney damage in a mouse model of chronic fibrotic kidney injury [64,65,66]. These results suggest that the TGFβ–EMT axis could be used as a therapeutic target against fibrosis. The role of sirtuins in renal fibrosis has been shown for SIRT1 through oxidative stress suppression [67], as well as the inhibition of HIF-2α expression [68]. Protein hyperacetylation has been documented in tubular epithelial cells during renal fibrosis, an event leading to a reduction in mitochondrial metabolic and energetic dysfunction. In this case, the activation of mitochondrial sirtuin SIRT3 can reduce the hyperacetylation of mitochondrial proteins, restoring mitochondrial function and suggesting an important role for this sirtuin during the metabolic changes induced by renal fibrosis [69]. Finally, SIRT6 also plays an important role in maintaining kidney function, delaying the onset of acute and chronic kidney disease [70].

PULMONARY FIBROSIS: Lung epithelial cells that are repeatedly damaged and inflamed show signs of EMT, leading to fibrosis [71]. Although the origin of myofibroblasts in lung fibrosis is not certain, some studies have suggested a role for TGFβ signaling in EMT-mediated lung fibrosis [72]. Primary alveolar epithelial cells (AECs) cultured on provisional matrix components fibronectin or fibrin undergo EMT via the integrin-dependent activation of endogenous latent TGFβ1, indicating that the ECM acts as a regulator in the EMT process during fibrogenesis [73,74]. Bulk RNA-seq and scRNA-seq data have been used to identify 10 major cell types and 19 subtypes at the single-cell level, and of these, macrophages and monocytes showed the greater activity in terms of inflammatory responses. Wang J et al. noted that fibroblasts exhibited greater EMT activity. In their work, they noted that fibroblasts had the greatest regulatory potential for KRT17 base-like epithelial cells with increased expression levels of COL1A1, NID1, CYR61, ANGPTL1, and CFH, while macrophages, monocytes, and endothelial cells had significant regulatory potential for SPP1, PLAU, MMP9, and PLAT [75]. SIRT1 can delay the onset of pulmonary fibrosis by regulating inflammatory, apoptotic, and autophagic pathways [76]. TGFβ-induced lung fibrosis could also be prevented or reduced by SIRT3 increased expression and activation. This effect was obtained by treating lung fibroblasts with hexafluoro, a novel fluorinated synthetic honokiol analogue derived from magnolia-tree bark that increases SIRT3 expression, reducing ROS accumulation [77]. Importantly, the activation of SIRT6 could prevent experimentally induced idiopathic lung fibrosis by reducing EMT in lung epithelial cells. This effect was obtained through SIRT6, as well as through the repression of the TGFβ1–SMAD3 pathway [78]. Finally, SIRT7 expression also decreased during pulmonary fibrosis and the overexpression of SIRT7 is associated with reduced pro-fibrotic markers [79].

CARDIAC FIBROSIS: EMT plays a role in regeneration and fibrosis after heart damage. In fact, EMT produces mesenchymal cells that have both stem and myofibroblast characteristics. In the case of post-myocardial injury, epicardial-derived cells undergo EMT and migrate to the damaged myocardium, generating different cell types in vivo, including cardiac interstitial fibroblasts and coronary smooth muscle cells, which aid the heart repairing process [80]. TGFβ promotes epicardial cell transformation and smooth muscle differentiation. In fact, adult human epicardial cells with an epithelium-like phenotype expressing the cell-surface marker, vascular cell adhesion marker 1 (VCAM-1), were spontaneously subjected to EMT and adopted a smooth-muscle-like phenotype [81]. Interestingly, TGFβ signaling can be modulated by SIRT1, suggesting an additional role for this sirtuin in reducing cardiac fibrosis [76]. Cardiac fibrosis can also be prevented by SIRT3 by deacetylating target proteins. In fact, SIRT3 deacetylates and activates GSK3β, thereby preventing TGFβ/Smad3 profibrotic genes [82]. Other profibrotic pathways inhibited by SIRT3 are FOS/AP1 and STAT3-NFATc2 [83,84]. Interestingly, the hearts of SIRT6 Tg mice have a reduced expression of fibrotic markers, suggesting a role for this sirtuin in delaying aging-induced cardiac fibrosis [85].

HEPATIC FIBROSIS: Chronic liver disease gives rise to hepatic fibrosis, but the origin of the activated myofibroblasts is still under debate, and various epithelial cells undergoing EMT may serve as sources. Hepatic stellate cells (HSCs), such as epithelial hepatocytes and cholangiocytes, are possible candidates to contribute to the myofibroblast population in hepatic fibrosis [86]. Again, TGFβ may be involved in the induction of the EMT phenotype in liver fibrosis, via the activation of the TGFβ1/SMAD pathway [87]. The role of SIRT1 in preventing liver fibrosis mainly involves the prevention of redox stress and inflammation by deacetylating and inhibiting NF-kB, PPAR-γ, etc. [76]. Liver fibrosis was also observed in SIRT2 KO mice, suggesting a role for this deacetylase in preventing profibrotic molecular pathways [88]. SIRT6 can also prevent liver fibrosis by deacetylating SMAD2/3, an event that is prevented by the accumulation of the pro-fibrotic protein, S100A11 [89].

SCLERODERMA AND SKIN FIBROSIS: Scleroderma (Sc) is a systemic disorder characterized by autoimmunity, chronic inflammation, vasculopathy, and extensive skin and organ fibrosis of unknown etiology [90]. In this disease, early vascular damage precedes fibrosis, and TGFβ induces myofibroblast gene expression with the production of pro-fibrotic cytokines and proteases [91]. In Sc epidermis, keratinocytes activate TGFβ/SMAD signaling with increased expression of connective tissue growth factor (CTGF) and SNAIL1 [92]. A link between unresolved inflammation and features of EMT during fibrogenesis in hypertrophic scar tissue has been suggested, with an increased expression of the mesenchymal marker FSP1 observed along with the EMT proteins SNAIL2 and TWIST [93]. Interestingly, decreased SIRT1 and SIRT3 serum levels have been measured in scleroderma patients compared to controls [94]. Moreover, SIRT1 has been shown to reduce skin fibrosis through TGFβ/Smad3 inhibition [95].

### 2.2. Cancer

Cancer-associated EMT is a complex and comprehensive form of cellular reprogramming, involving metabolic, epigenetic, and morphological changes. Growing evidence has revealed the functional association between EMT and cancer stem cells (CSCs) [96]. CSCs are regarded as a small subset of self-renewing tumor cells that have the capacity to initiate and sustain tumors. CSCs are highly tumorigenic and metastatic, and they are responsible for tumor resistance to cancer therapies [97].

#### 2.2.1. EMT Regulators and Cancer Stem Cells

Several studies have shown that EMT regulators are closely linked to the induction of stem-cell properties in many human cancers [98,99]. Considering EMT-related proteins, Twist1 and Twist2 contribute to overcoming the initial barrier to tumor development by inhibiting Ras-induced premature senescence [100]. Similarly, ZEB proteins prevent EGFR-induced premature senescence in esophageal carcinogenesis [101]. Furthermore, the Wnt/β-catenin pathway induces the expression of SLUG1 and TWIST1 [102], thereby contributing to CSCs formation. These findings suggest that EMT has a role in generating stem-like cells, and may contribute to resistance to anti-cancer therapy.

#### 2.2.2. EMT Regulators and Tumor Metastasis

EMT regulators have also been associated with tumor progression and metastasis. For example, SNAIL1 expression was associated with distant metastasis in various human cancers, including breast, ovarian, and squamous cell carcinomas [103], while SLUG1 promotes tumor invasion in lung and colon carcinomas, as well as the bone marrow homing of leukemic cancer cell lines. A mechanistic link between TWIST1, ETM, and metastasis has been established in human breast cancers [104]. In addition, vimentin is over-expressed in many epithelial cancers, including lung cancer, and its overexpression correlates with tumor growth, invasion, and poor prognosis [105]. NOTCH is implicated in the acquisition of EMT and invasion in pancreatic cancer cells [106].

#### 2.2.3. EMT-Associated Transcription Factors and miRNA

The EMT-associated transcription factors also regulate non-coding RNA expression. EMT factors may also induce the transcriptional repression of let-7 and other tumor-suppressor miRNA families. TWIST can repress let-7 transcription, thereby freeing the expression of stem factors such as Oct4, Sox2, Klf4, and c-Myc [107]. Another miRNA repressing EMT, miR-34, is part of an interesting double-negative feedback loop with SNAIL1. In normal cells, miR-34 represses SNAIL1 and EMT. However, in cancer cells, the upregulation of SNAIL1 inhibits miR-34 expression, inducing EMT [108]. This double-negative feedback loop is also common in miR-200, another important negative regulator of the transcription factors associated with EMT in cancer [109]. In fact, miR-200 inhibits the expression of ZEB1, while ZEB1 represses miR-200a, miR-200b, and miR-200c promoters [110]. These findings underline the presence of an altered homeostatic equilibrium among EMT transcription factors and miRNAs in cancer and suggests, at the same time, that restoring or pushing cancer cells away from it may represent a new anti-tumor strategy.

## 3. Hypoxia-Induced EMT

Hypoxia is recognized as a hallmark of cancer and the activation of hypoxic responses is a common characteristic of many tumors [111,112].

A large number of studies have demonstrated the strict connection between hypoxia and EMT or fibrosis. However, it is important to underline that the hypoxic microenvironment is created during the phase of tumor progression when tumor cells’ growth outpaces neo-vascularization and the tumor increases its volume to 1 mm or more. Subsequently, hypoxia greatly contributes to the acquisition of new properties by the tumor cells, such as neoangiogenensis, metabolic reprogramming, immune-cell hijacking, EMT induction, and tissue invasion, to name a few. Therefore, hypoxia-induced EMT is more closely linked to a secondary and, for the patient, more devastating phase of the tumor history compared with the initial transformation phase, namely tumor progression and metastasis formation.

**Figure 4:**
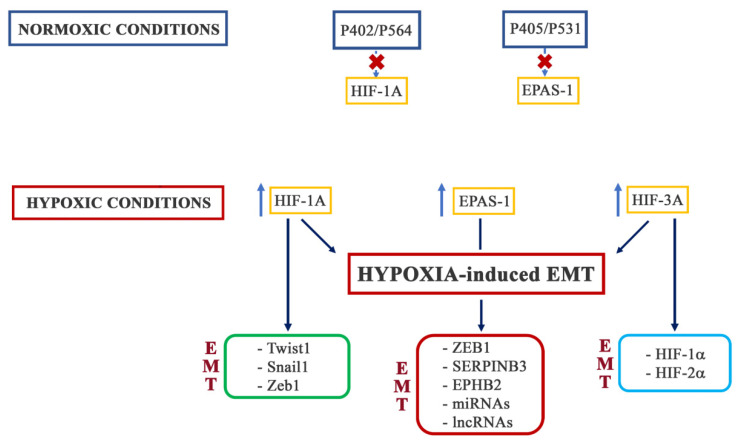
Regulation of EMT by hypoxia through the induction of HIF-1, EPAS-1 (HIF-2), and HIF-3 transcription factors (HIF-dependent EMT induction). HIF-1 and HIF-2 directly regulate transcription factors directly or indirectly involved in EMT. On the other hand, the little that is known about HIF-3 suggests control over HIF-1α and HIF-2α more than a direct effect on EMT-transcription factors.

Due to the critical role of oxygen for cellular activity, cells have developed physiological adaptation processes through the activation of the hypoxia-inducible factors (HIFs) [111,112]. As detailed in a large number of reviews and articles, included in the references below, HIFs are heterodimer DNA-binding complexes consisting of two basic helix–loop–helix proteins of the PAS family [113], a constitutively expressed HIF-1α subunit, and an oxygen-regulated HIFα [114]. In mammals, the alpha subunits are encoded by three different genes: HIF-1A, EPAS1 (also known as HIF-2A), and HIF-3A [115,116,117,118,119,120]. The different ways through which hypoxia can activate EMT are schematized in Figure 4.

### 3.1. HIF-1

HIF-1α regulates the expression of several EMT-TFs, such as Twist1, Snail1, and Zeb1, among others, participating in EMT-associated signaling pathways. By binding to the HRE in the proximal promoter of TWIST1, HIF-1α directly upregulates its expression. This activity has been observed in several cancer models, including, but not limited to, hepatocellular carcinoma, ovarian cancer, and non-small-cell lung cancer [121,122]. Instead, several mechanisms have been proposed for the HIF-1α-induced expression of SNAIL1. Besides the binding of the HRE sequences in the SNAIL1 promoter [122], HIF-1α can reduce the proteasomal degradation of Snail1 by inducing the activity of the deubiquitinating enzyme ubiquitin-specific protease 47 (USP47) [123]. Moreover, the induction of HDAC3 activity by HIF-1α has also been shown to increase Snail1 expression [124]. Finally, through the induction of the TGFβ pathway, HIF-1α participates in SNAIL1 expression [125]. Much like TWIST1 and SNAIL1, the ZEB1 promoter region contains an HRE that is recognized and directly bound by HIF-1α [126,127]. Recently, it was reported that HIF-1α could also bind to the promoter region of ZEB2 [128]. In lung and pancreatic cancer and in head-and-neck squamous carcinoma, HIF-1α has been suggested to induce the expression of Slug through the binding of HRE in its promoter [129]. Interestingly, HIF-1α could act on some EMT-TFs indirectly. In prostate cancer, HIF-1α induced an increased expression of Forkhead box M1 (FoxM1) by binding to its promoter [130], while in pancreatic cancer, HIF-1α promoted PAFAH1B2 expression [131].

### 3.2. HIF-2

Although HIF-2α shares a 48% similarity in amino acid sequence with HIF-1α and has a similar domain arrangement, it has different expression levels in different tissues during developmental stages and shows different specificity in its transcriptional targets [132].

Several studies have demonstrated that HIF-2α promotes the expression of EMT markers. In colorectal cancer (CRC), HIF-2α, by binding to the HRE sequences in the ZEB1 promoter, induced its activity and regulated EMT through the downregulation of HINT2 [133]. In pancreatic cancer, HIF-2α promoted the EMT process through the regulation of the binding of Twist2 to the promoter of E-cadherin [134]. Moreover, in liver cancer cells, it has been observed that hypoxia, through a HIF-2α-dependent mechanism, upregulated the transcription, synthesis, and release of SERPINB3, a molecule that has been correlated with the EMT process [135]. In glioblastoma, HIF-2α promoted the expression of EPHB2, a protein involved in the EMT process in epithelial cancer cells through the phosphorylation of paxillin [136]. In pancreatic cancer, HIF-2α transcriptionally activated miR-301a by targeting TP63-induced EMT [137], while in clear-cell renal carcinoma cells, the long non-coding RNA LINC01234 was involved in HIF-2α-mediated hypoxia-induced EMT [138]. In hepatocellular carcinoma (HCC) and non-small-cell lung cancer (NSCL), the EMT process was promoted by the overexpression of the long-non-coding RNA NEAT1 induced by HIF-2α [139,140]. Furthermore, in lung cancer, HIF-2α also induced EMT by increasing the expression and stabilization of β-catenin with the consequent activation of Wnt signaling [141]. An interesting study by Sun et al. demonstrated that in HCC, the activity of HIF-2α and, therefore, the induction of EMT, was regulated by Acetyl-CoA synthase 2 (ACSS2). ACSS2 played an important role in the acetylation of HIF-2α, and its knockdown under hypoxic conditions reduced the acetylation levels of HIF-2α, enhancing the expression levels of its downstream molecules [142]. Interestingly, it has been shown that the hypoxia-associated factor (HAF), which is often overexpressed in a variety of tumors following prolonged hypoxia, was responsible for switching the hypoxic response from a HIF-1α-dependent to a HIF-2α-dependent transcription. This process was correlated with the activation of the genes involved in EMT, such as MMP9, PAI-1, and the stem-cell factor OCT-3/4 [143].

### 3.3. HIF-3

HIF-3 is the least understood of the HIF transcription factors. This is partly due to the existence of multiple HIF-3α variants. Indeed, the HIF-3 gene contains three alternative promoters and generates multiple mRNA through the utilization of different transcription initiation sites and alternative splicing [144]. It was originally thought that HIF-3α could have an inhibitory effect on HIF-1α and HIF-2α transcriptional activity and, since, the expression of the HIF-3 gene is induced by HIF-1α, this suggested the existence of a possible negative-feedback mechanism to attenuate HIF-1α activity during prolonged hypoxia [145]. It is now known that some HIF-3α variants can inhibit HIF-1 and 2α action by competing for binding to HIF-1-β. Moreover, other truncated variants act as dominant-negative regulators of HIF-1 and 2α. However, there are still several HIF-3αvariants without functions. In addition to this inhibitory function, HIF-3αactivates a unique transcriptional program that, in zebrafish, resembles that of HIF-1α [146]. However, to date, there is no information about the possible direct involvement of HIF-3α in the hypoxia-induced EMT process.

## 4. HIF-Independent Hypoxia-Induced EMT

Hypoxia can also regulate other HIF-1 independent signaling pathways that are recognized as EMT inducers, particularly NF-kB, TGFβ, and Notch.

### 4.1. NF-kB

NF-kB is a family of transcriptional factors comprising different proteins such as RelA, RelB, cRel, NF-kB1 (p105/p50), and NF-kB (p100/p52), which are confined in the cytoplasm by the inhibitor of kB (IkBs) [147]. Once the NF-kB signaling is initiated, mainly following the activation of the receptor belonging to the TNF (TNFR), Toll-like (TLR), and pattern-recognition receptor (PRR) families, NF-kB translocates into the nucleus and stimulates a variety of cell processes, including proliferation, inflammation, and EMT [148,149]. Indeed, the activation of NF-kB, induces the expression of TWIST1, SNAIL2, and ZEB2, by binding to their promoter regions [150], and of SNAIL1, by upregulating COPS2, which is responsible for blocking SNAIL1 ubiquitination and degradation [150]. However, the activation of NF-kB under hypoxic condition is still an object of debate. The first indications suggested that hypoxia-induced NF-kB is mediated by the phosphorylation of a tyrosine residue on IkBα, which is responsible for the dissociation and degradation of this inhibitor [151]. Recently, it was hypothesized that PHDs could have a role in the control of the inhibitor of the kB kinase complex (IKK), which, by phosphorylating IkBs, determines its dissociation from NF-kB, thus allowing the translocation of NF-kB to the nucleus. However, it is still not clear whether the enzymatic activity of the PHDs is required in this process [152]. In addition, the hypoxia asparaginyl hydroxylase, known as factor-inhibiting HIF (FIH), has also been proposed as a possible actor in the regulation of NF-kB under hypoxia due to its ability to post-translationally modify multiple components of the NF-kB pathway [153]. Another mechanism that has been shown to activate NF-kB under hypoxia involves the transforming growth factor activating kinase 1 (TAK1) a member of the MAPK family. In particular, the calcium released from cellular compartment following hypoxia triggers CamK2, which, in turn, activates TAK1. Subsequently, the complex TAK1-TAB binds XIAP, leading to IKK activation [154].

### 4.2. TGFβ, MAPKs and mTOR

Under hypoxic conditions, TGFβ production is significantly enhanced, suggesting a possible mechanism by which hypoxia may modulate EMT [155,156,157]. The non-SMAD signaling pathway involves various MAPKs and the PI3K-Akt-mTOR pathway [157]. The PI3K-Akt-mTOR pathway has an important role in the induction of EMT, mainly through Snail and Slug upregulation [158,159], and is one of the crucial pathways involved in the hypoxia response. Interestingly, PI3K-Akt-mTOR could influence the translation of HIF-1α via the co-operative regulation of both initiation factor 4E-binding protein 1 (4E-BP1) and ribosomal protein S6 kinase-1 (S6K1) [160]. JNK is the only MAPK whose participation in hypoxia-induced EMT has been reported [161].

### 4.3. Notch, AMPK

The Notch-signaling pathway is another signaling pathway involved in the EMT process that can be activated by hypoxia. Most of the data regarding the hypoxia-induced activation of Notch have been observed in tumor cells. In breast cancer cells, it has been demonstrated that Notch can directly induce Slug, but not Snail and Twist1 [162], while in prostate cancer, Notch was associated with Snail and Zeb1 [163]. Both HIF-1α and HIF-2α have been reported to synergize with the Notch co-activator MAML1 in promoting Notch activity [164]. Hypoxia has also been demonstrated to activate the AMP-activated protein kinase (AMPK), an evolutionarily conserved stress-sensing kinase [165]. AMPK has a controversial role in the context of EMT. Indeed, in some tumors, the activation of this signaling pathway has been correlated with an increase in the expression and nuclear localization of TWIST1 [166], while in other studies, AMPK activation was linked to the suppression of the EMT process [167,168]. In particular, Chou et al. demonstrated that EMT was suppressed by the modulation of the Akt-MDM2-Foxo3 signaling axis induced by AMPK activation [169]. It is worth nothing that in all the aforementioned studies, the activation of the AMPK was induced by treatments different from hypoxia; however, it is conceivable that low oxygen tension could produce similar results.

### 4.4. Microenvironment

In the last year, it has been proposed that the modulation of the microenvironment induced by hypoxia could affect EMT, mainly through the remodeling of the extracellular matrix and the regulation of immune cells. As previously described, the role of ECM in the modulation of EMT has long been recognized [170]. It is therefore conceivable that the effects of hypoxia on ECM could affect hypoxia-induced EMT. Hypoxia can regulate different components of the ECM, such as collagen I and fibronectin, and is responsible for changes in its composition and organization through the modulation of matrix metalloproteases (MMPs) [171]. Moreover, hypoxia can influence the expression of integrins and cadherins, which are classically involved in the EMT process. It has been observed that in tumor microenvironment, a reduced infiltration of immune cells and the presence of suppressive or exhausted immune cells might be required to facilitate the process of EMT [172]. Hypoxia could induce changes in the activity and function of immune cells, such as increasing the secretion of the EMT-inducing cytokines TNFα, CCL2, IL-1, and IL-6 from macrophages [173], but it may also promote a permissive immune environment [174].

### 4.5. microRNA

Hypoxia can also induce EMT by regulating microRNA (miRNA). In particular, in the tumor hypoxic niche, hypoxia suppresses DICER, which is responsible for processing microRNA precursors (pre-miRNAs) into mature miRNAs, through an epigenetic mechanism that involves the inhibition of oxygen-dependent H3K27me3 demethylases KDM6A/B [175]. Moreover, recently, it was demonstrated that Nut77, whose expression is induced under hypoxic conditions, plays an important role in the regulation of miRNA biogenesis during hypoxia-induced EMT [176]. So far, the following, miRNAs have been linked to the hypoxia-induced EMT process: Mir-204 [177], Mir-1236 [178], Mir 210-3p [179], miR-545-3p-TNFSF10 [180], mir200a [181], miR-1296 [182], miR-3194– 3p [183], and miR-34a [184].

**Figure 5:**
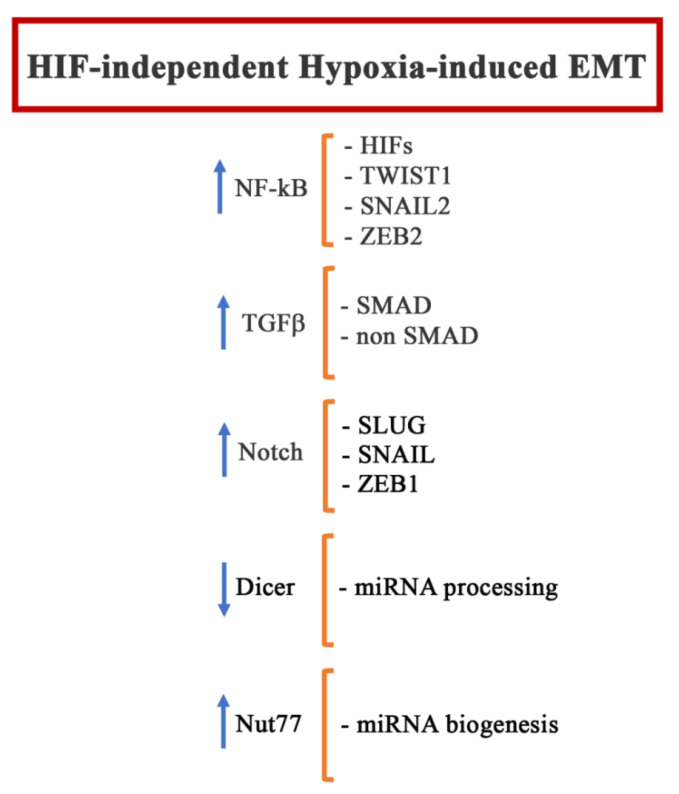
Regulation of EMT-related actors by hypoxia in a HIF-independent way. Transcription factors (NF-kB), growth factors (TGFβ), membrane receptors (Notch), and miRNAs can be modulated during hypoxia to control EMT.

Figure 5 summarizes the main EMT players modulated in a HIF-independent way during hypoxia.

## 5. Sirtuins in EMT

**Figure 6:**
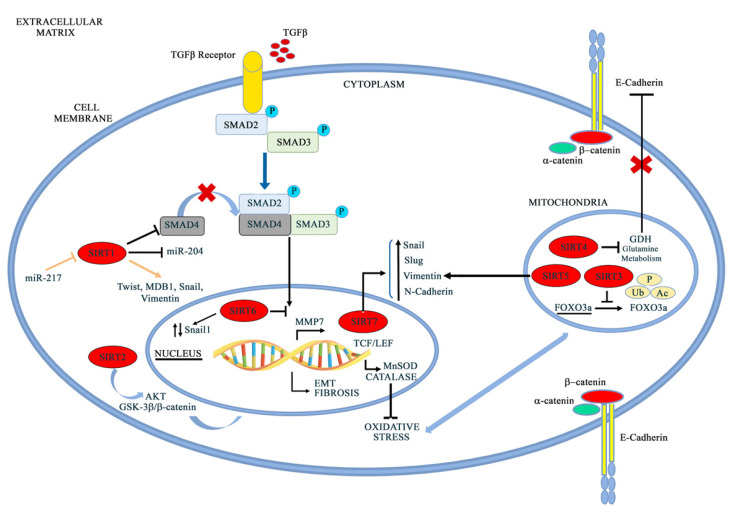
Schematic illustration of the molecular mechanisms of Sirtuins in regulating EMT. The scheme illustrates cytosolic (SIRT1 and SIRT2), nuclear (SIRT6 and SIRT7), and mitochondrial (SIRT3, SIRT4, and SIRT5) sirtuins. Some important sirtuins target involved in EMT are indicated, such as SMAD4, which is released upon SIRT1 downregulation, increasing nuclear accumulation of β-catenin and EMT induction. Similarly, SIRT2 increases nuclear β-catenin by deacetylating AKT. Nuclear sirtuins, such as SIRT6 and SIRT7, increase the activity of EMT transcription factors, such as Snail, Slug, and Vimentin. On the other hand, the role of mitochondrial sirtuins is still controversial, with some reports indicating the pro-EMT action of SIRT3 through an increase in β-catenin, while others have shown the anti-EMT action of SIRT3 through the repression of β-catenin and Twist1. Very little is known about SIRT4 and SIRT5, although they seem to prevent EMT induction.

Sirtuins are a class of enzymes that are highly conserved from yeast to humans [185]. Seven mammalian sirtuins have been identified (SIRT1–SIRT7), characterized by different cellular localization, functions, and structure [186]. Although these enzymes share a catalytic core domain and a NAD+ binding domain, they also present some differences in their sequence that are responsible for their intracellular function and localization, as well as for their target specificity [187]. Sirtuins were first characterized as histone deacetylases (HDACs), but subsequent and more detailed studies have underlined the presence of non-histonic targets [188]. According to their cellular localization, sirtuins are classified as nuclear (SIRT1, SIRT6), nucleolar (SIRT7), mitochondrial (SIRT3, SIRT4, SIRT5), and cytoplasmatic (SIRT2) [189]. Sirtuins can change their localization following different stimuli. SIRT1 can shuttle to the cytoplasm [190], SIRT2 migrates to the nucleus during the G2/M phase of the cell cycle [191], and SIRT3 localizes to the nucleus in the presence of stressful conditions [192]. This class of proteins has drawn attention because of its involvement in many different physiological processes, such as aging and longevity [193], stress response, inflammation, and metabolism [194], as well as in several pathological conditions, such as neurodegenerative disorders [195,196], cardiovascular diseases [197], metabolism-related disorders [198], carcinogenesis, and tumor development [199], in which sirtuins can act as disease promoters or protective factors [200]. In fact, over the past decade, several studies have been conducted in order to investigate the role of sirtuins in physiological and pathological conditions, such as cancer [201,202]. The role of different sirtuins in regulating EMT is represented in Figure 6 and extensively described below.

### 5.1. SIRT1

Several studies have highlighted the role of SIRT1 in EMT activation or repression based on different cancer histotypes. SIRT1 reduction promotes EMT, metastasis, and tumor progression in breast and kidney epithelial cells associated with TGFβ hyperactivation and Smad4 hyperacetylation. Furthermore, a decrease in SIRT1 expression causes an overexpression of MMP7, a Smad4 target, which, in turn, leads to E-cadherin degradation, thereby releasing β-catenin from the cadherin junctions. In this way, β-catenin migrates to the nucleus, enhancing the EMT process [203]. Conversely, in prostate cancer, SIRT1 causes an increase in cell migration and metastasis, physically interacting with the zinc finger transcription factor ZEB1, thus suppressing E-cadherin transcription through histone H3 deacetylation in the E-cadherin proximal promoter [204]. Deng et al. directly connected SIRT1 overexpression with chronic pancreatitis and pancreatic cancer and subsequently evaluated the role of SIRT1 and miR-217 in EMT progression. They first demonstrated the ability of miR-217 to negatively regulate the 3′UTR of SIRT1, causing a decrease in SIRT1 mRNA translation; in the same study, SIRT1 knockdown was strictly connected with mesenchymal-to-epithelial transition (MET) in pancreatic cancer cells [205]. The course of pancreatic cancer also relies on methyl-CpG binding domain protein 1 (MBD1), which, when upregulated, correlates with lymph node metastasis and poor prognosis for patients [206]. In fact, this protein is associated with TWIST and SIRT1, thus creating a complex that is able to decrease E-cadherin transcription activity with EMT induction [207]. SIRT1 is also upregulated in hepatocellular carcinomas (HCCs), where it increases cancer cells’ invasiveness and metastatic potential through the activation of SNAIL, TWIST, and VIMENTIN, and decreases the expression of E-cadherin [109,207]. Furthermore, in gastric cancer, SIRT1 negatively regulates miR-204, promoting cell invasion connected with the inactivation of LKB1. In this situation, miR-204 acts as a tumor suppressor, while SIRT1 downregulation induces a MET phenotype, with a decrease in Vimentin and an increase in E-cadherin expression [208]. SIRT1 is highly expressed in metastatic melanoma, where it accelerates EMT through Beclin1 deacetylation, enhancing E-cadherin autophagic degradation [209]. In breast cancer, a SIRT1-PRRX1-KLF4-ALDH1 circuitry has been identified, in which SIRT1 deacetylases and stabilizes the epithelial-to-mesenchymal-transition inducer, PRRX1, by inhibiting its proteasomal degradation [210].

### 5.2. SIRT2

SIRT2 is involved in EMT in hepatocellular carcinoma (HCC). Chen et al., in fact, reported that the upregulation of SIRT2 in HCC tumors was correlated with the presence of vascular invasion, a higher tumor stage, and a shorter overall survival. This evidence underlined the role of SIRT2 in cell motility and invasive phenotypes, markers of EMT process. In the specific context of hepatocellular carcinoma, SIRT2 regulates Akt deacetylation and activity, thus promoting glycogen synthase kinase-3β (GSK-3β)/β-catenin signaling and causing EMT and cell migration [211]. The possible involvement of SIRT2 in EMT is corroborated by its activity in colon cancer cells. Treatment with benzylsulfonamide AK-1, a SIRT2 specific inhibitor, induces Snail proteasomal degradation. Snail reduction causes p21 upregulation with consequent cell-cycle arrest, slow proliferation, and impaired wound-healing activity [212].

### 5.3. SIRT3

Among mitochondrial sirtuins, SIRT3 has an important role in EMT regulation and metastatic motility in several cancer phenotypes. In a preliminary study on prostate cancer, Li et al. found a tight connection between the SIRT3-FOXO3A axis and the Wnt/β-catenin pathway [213]. FOXO3A is, in fact, a downstream target gene of SIRT3 [214], and these two factors physically interact, causing the nuclear localization of FOXO3A and, in turn, the transcription of FOXO3A-dependent genes [215]. In particular, FOXO3A regulates PUMA expression with apoptosis induction and EMT repression [216]. On the other hand, SIRT3 inhibits the Wnt/β-catenin pathway and, indirectly, Twist1, Twist2, and Snail2, which are tightly connected with EMT progression, cancer invasion, and metastasis [217]. Other studies conducted on ovarian cancer have underlined SIRT3 downregulation in the metastatic tissues and highly metastatic cell lines of ovarian cancer [218]. In these studies, SIRT3 inhibits ovarian cancer metastasis by repressing Twist expression, thereby impairing EMT progression [218]. SIRT3′s protective action against EMT and tumor progression was also found in renal tubule interstitial fibrosis, the hallmark of chronic kidney disease, characterized by a significant rate of EMT strictly connected with Angiotensin II expression [219]. Angiotensin II (ANG II), in fact, induces EMT in a TGFβ-dependent and -independent manner [220,221]. Furthermore, oxidative stress is recognized as an important mediator of kidney damage and kidney pathogenic injuries [222,223]. In this scenario, SIRT3 plays an important role through its activity on its downstream mediator MnSOD, which is involved in mitochondrial protection against ROS. Recent studies, in fact, demonstrate that mitochondrial damage and ROS production increase aldosterone-induced EMT [224], but in a more detailed manner, SIRT3 ameliorates the pathogenesis of renal tubular EMT by blocking ANG II action on mitochondrial degeneration. A decrease in SIRT3 expression results in a decline in the mtDNA copy number, a reduction in the mitochondria number, and disrupted mitochondrial morphology, with consequent mitochondrial dysfunction [219].

### 5.4. SIRT4 and SIRT5

Among mitochondrial sirtuins, less is known about the involvement of SIRT4 and SIRT5 in the EMT process. Nevertheless, SIRT4 is connected to the increase in E-cadherin expression, preventing proliferation, migration, and invasion in colorectal cancer cells through glutamine metabolism inhibition [225]. On the other hand, Guoet et al. demonstrated that SIRT5 suppresses EMT in hepatocellular cancer (HCC) through Snail downregulation and E-cadherin upregulation. Furthermore, SIRT5 directly binds Vimentin, which, in more metastatic HCC phenotypes, shows an acetylation site on K120, which could be a target for SIRT5 activity, enhancing the metastatic process in HCC [225].

### 5.5. SIRT6

Recent studies have underlined the role of SIRT6 in the development of several types of cancer, such as breast cancer, liver cancer, pancreatic cancer, and colon adenocarcinoma [226,227,228]. Among SIRT6 interactors, Snail represents a point of connection between SIRT6 and EMT in colon carcinoma cells, although the deacetylation site on Snail protein is unknown [229]. Geng et al. also demonstrated that SIRT6 can suppress the transcription of TET1, a tumor suppressor in several cancer types, promoting the EMT process through its H3K9 deacetylase activity [229]. Several other cellular systems have been analyzed to elucidate the connection between SIRT6 and Snail. Li et al., in non-small-cell lung cancer (NSCLC) cells, demonstrated that SIRT6 silencing causes a reduction in Snail protein [230]. This evidence suggests that SIRT6 may be involved in EMT through post-translational modifications to Snail protein. In fact, several data obtained by the same research group underlined the role of SIRT6 in enhancing Snail protein stabilization through its deacetylation and regulation of the promoter site of KLF4, a downstream target of the Snail signaling pathway [231]. Furthermore, SIRT6 enhances the EMT process in hepatocellular cancer (HCC), promoting E-cadherin autophagic degradation in a Beclin-1 dependent manner [231].

### 5.6. SIRT7

SIRT7 has been implicated in EMT modulation through the downregulation of N-cadherin and vimentin and the upregulation of E-cadherin expression in oral squamous cell carcinoma (OSCCs) via SMAD deacetylation [232,233]. SIRT7 is also involved in Snail and Slug upregulation, which, in turn, reduces E-cadherin and increases N-cadherin and vimentin [234]. Alternatively, SIRT7 directly binds the E-cadherin promoter, deacetylating H3K18. This reaction causes the inhibition of E-cadherin transcription in a chromatin-remodeling manner [235]. A connection between SIRT7 and EMT has been demonstrated in bladder cancer, where SIRT7 silencing decreased E-cadherin expression and increased EMT markers, such as N-cadherin, Slug and Snail [236]. Conversely, in prostate cancer, SIRT7 depletion impairs migration and invasiveness, restoring an epithelial gene expression and a non-pathological phenotype [237]. These different effects of SIRT7 on different tissues may be due to the wide range of its activity and to the dissimilar molecular profiles of the studied cancers. Furthermore, Monteiro-Reis et al., in their study on bladder cancer, revealed a connection between SIRT7 and EZH2, members of the polycomb repressive complex 2 (PRC2), which is involved in transcription repression on several gene promoters. When SIRT7 is downregulated, EZH2 is acetylated and active, causing a shift from the epithelial to the mesenchymal phenotype, with an increase in cancer cell motility [236]. In this cancer, SIRT7 plays a dual role. An increase in SIRT7 expression is fundamental for urothelial neoplastic transformation, resulting in a non-invasive urothelial carcinoma in situ characterized by cell growth, high cell survival, and the deacetylation of H3K18, strictly connected with neoplastic development [238,239]. The transition to a more invasive and aggressive phenotype instead requires SIRT7 downregulation and involves EZH2 upregulation and acetylation in order to promote EMT [236]. These data, taken together, underline SIRT7’s plasticity during carcinogenesis and tumor progression.

## 6. Sirtuins in Hypoxia

Sirtuins influence hypoxia and hypoxia-induced EMT by modulating the activity of different transcription factors. Table 1 summarizes the role of sirtuins in regulating HIF-1α, HIF-2α, and EMT-related proteins in the context of cancer.

### 6.1. SIRT1

SIRT1 deacetylates HIF-1α and HIF-2α, with opposite effects [240,241,242]. SIRT1 represses Notch and mitigates Wnt-dependent EMT deacetylating β-catenin [246,247], exerting a negative check on EMT-related pathways [243]. Sun et al., demonstrated that the SIRT1 repression of ovarian cancer development and EMT can be prevented by the SUMOylation-and degradation of this sirtuin [244]. In this cancer, SIRT1 deacetylates HIF-1α at lysine 674 inhibiting the interaction between this transcriptional factor and p300, thereby maintaining an epithelial-like phenotype [240]. On the other hand, SIRT1 enhances HIF-2α activity through its deacetylase activity, inhibiting cancer metastasis formation [245].

### 6.2. SIRT2

SIRT2 prevents the expression of HIF-1α protein and the related hypoxia-dependent genes, such as VEGF [248]. SIRT2 regulates HIF-1α stability, increasing deacetylation and hydroxylation. In particular, an increase in the NAD+/NADH ratio causes HIF-1α degradation in a SIRT2-dependent manner [252]. To underline SIRT2’s role in HIF-1α stability, several studies demonstrated that SIRT2 deacetylates the NF-kB p65 subunit, inhibiting the expression of NF-kB-dependent genes. This suggests the involvement of NF-kB suppression in HIF-1α expression mediated by SIRT2 activity [252]. HIF-1α regulation, mediated by SIRT2, occurs at protein and not at mRNA level, through Lys709 deacetylation. This lysine residue represents an important site of acetylation and ubiquitination [249]. In this way, SIRT2-mediated HIF-1α deacetylation leads to its hydroxylation and degradation. Significantly, HIF-1α degradation, which is connected to SIRT2 activity, underlines the tumor suppressor role proposed for SIRT2 in many studies [250,251].

### 6.3. SIRT3

SIRT3 is a mitochondrial sirtuin, described as a tumor suppressor protein for its ability to suppress ROS generation [253]. Among SIRT3 targets, MnSOD and IDH2, localized in the mitochondrial matrix, have been demonstrated to inactivate HIF-1α by reducing ROS accumulation [253]. The same study found that SIRT3 decreases HIF-1α protein stabilization through the ROS-mediated alteration of hydroxylation and proteasomal degradation [254]. In this way, HIF-1α cannot activate the transcription of genes involved in angiogenesis, metastasis, and glycolysis [255].

### 6.4. SIRT6

SIRT6 downregulates the basal transcription of HIF-1α target genes in physiological conditions [256,257] through a direct interaction. Several studies have demonstrated that HIF-1α activates transcription through the recruitment of p300/CBP, underlining the opposite role played by SIRT6, which can compete with HIF-1α for p300 recruitment by maintaining the histones localized in its promoter sequence in an hypoacetylated status [256,258,259]. Further studies demonstrated that SIRT6 regulates HIF-1α activity at chromatin level by deacetylating H3K9 site in HIF-1α target genes [256]. To support this hypothesis, SIRT6 deficiency has been shown to promote HIF-1α protein synthesis and stability [256]. In this way, the deacetylation of acH3K9 suppresses HIF-1α activity with a reduction in glucose uptake, glycolysis, and an increase in oxidative phosphorylation [260]. By contrast, Young et al. determined that, in papillary thyroid cancer (PTC), increased SIRT6 stabilizes HIF-1α, activating EMT and cancer progression [261].

### 6.5. SIRT7

SIRT7 binds to both HIF-1α and HIF-2α, reducing their stability and their transcriptional activity. However, this effect occurs independently of SIRT7 deacetylase activity or of HIF-1α degradation pathways [262].

## 7. Pharmacological Control of EMT through Sirtuins and HIF Modulation: Nanomaterials and Nanomedicine

As previously discussed, the biology of the sirtuins family and HIF-1 is strictly interconnected with EMT regulation in cancer cells. Hence, recent studies proposed that targeting this oncogenic interplay may open new therapeutic avenues to treat cancer.

Lamentably, therapeutic nucleic acids (as gapmers, antisense oligonucleotides, and siRNAs [263]) and other chemotherapeutics, when administered alone, share some properties, including rapid biodegradation, poor solubility, and a lack of target specificity, with increasing side effects that have greatly limited their application in in vivo systems and in clinical applications [264].

To overcome these limitations, in the last decades, nanomaterials have been exploited for the systemic delivery of anticancer agents to allow their accumulation in tumor sites through the enhanced permeability and retention effect (EPR), which works because of the leaky tumor vasculature and poor lymphatic drainage [265]. Hence, the improvements in materials sciences have generated nanomaterials capable of overcoming biological barriers and able to transport anticancer agents to targeted sites while minimizing harmful effects on healthy tissues [266].

In this regard, different nanomaterials have been described as delivery scaffolds of nucleic acids or drugs modulating the intracellular levels of HIF-1 and sirtuins, which, in turn, may also lead to changes in the EMT process. These novel therapeutic approaches have attracted considerable interest as a novel tool for EMT modulation in the context of anticancer therapy [267].

**Figure 7:**
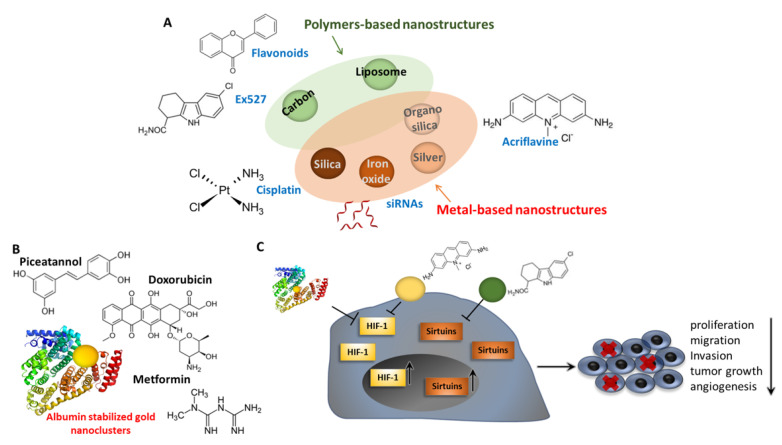
Schematic illustration of how different nanomaterials have been used or could be used to deliver nucleic acids or drugs to modulate hypoxic response and/or sirtuins in order to modulate the EMT process. (**A**,**B**) Modulators of sirtuins and/or HIF, such as natural compounds (Piceatannol, Flavonoids), drugs (Metformin, Doxorubicin, Cisplatin), and antibiotics (Acriflavine), could be delivered by **polymer-based nanostructures**, such as liposomes, **Albumin stabilized gold nanoclusters** or **metal-based nanostructures**, such as gold nanoclusters, iron oxide, etc. (**C**) pathways activated by hypoxia or sirtuins leading to EMT activation could be inhibited, preventing cancer-cell proliferation, tumor growth, invasion, migration and angiogenesis.

However, although many nanomaterials functionalized with different therapeutics have been shown to directly modulate EMT [267], it should be remembered that nanomaterials carrying molecules interfering with both sirtuins and HIF-1 may lead to antitumor activity in many other ways, due to their implication in multiple biological processes, as described above. For example, such nanostructures could lead to anticancer effects by activating autophagy or p53-dependent apoptotic cell death, as well as by eliciting other pathways directly related to the inhibition of cell proliferation and tumor vascularization and growth, as summarized in Figure 7 and described below. Overall, the effect of these nanomaterials on EMT may be indirect, by affecting closely related and interconnected pathways.

Different studies showed that novel polyethylene glycol-coated liposomes loaded with flavonoids enhance the anti-glioblastoma effect through the modulation of sirtuins. Interestingly, these nanostructures were shown to downregulate SIRT1, but also to increase the SIRT3/p53 axis, depending on the nature of the flavonoid carried. However, increased apoptosis and reduced cancer-cell proliferation were observed in glioma cancer cells as consequences of treatment with these nanocomplexes [268,269]. These studies suggest that the anti-glioma effects exerted by flavonoid-loaded nanoliposomas were mediated by the modulation of the SIRT1/SIRT3 signaling pathways.

Drug-delivery systems based on albumin are being investigated extensively in cancer therapy due to their excellent properties as selective carriers in cancer cells, and are frequently employed in combination with different molecules and nanomaterials to generate albumin-based nanostructures (ABN). In particular, albumin-stabilized gold nanoclusters present remarkable properties for biological application, including in in vivo applications, such as low toxicity and excellent stability [270,271,272]. In this regard, it is worth mentioning Abraxane^®^ (albumin-bound paclitaxel), a FDA-approved nanomedicine formed by albumin nanoparticles containing encapsulated paclitaxel (PTX), which is indicated for the treatment of metastatic breast and pancreatic cancer and advanced non-small-cell lung carcinoma [273].

In this context, researchers have linked hyaluronic acid (HA)-carbon dot (CD) conjugates with albumin to generate BSA-HA-CDs nanoparticles [274]. This nanocomplex was suitable for the encapsulation of drugs such as DOX and Met, generating a highly efficient therapeutic system for hypoxic tumors. Interestingly, the expression of HIF-1α was significantly decreased when treated with DOX/Met/BSA-HA-CDs in hypoxia condition compared with the control group, which resulted in inhibited drug efflux and increased cellular DOX accumulation [274]. Hence, we hypothesize that the albumin-based complexes described in this work could also affect EMT process and the related phenotype, opening new avenues for cancer treatment.

In addition to ABN, metallic nanomaterials have also been thoroughly exploited for biomedical applications because their intrinsic chemical physical proprieties and reactivity cause cytotoxic effects, among others. However, metal-based nanostructures are also used as nanocarriers for therapeutic molecules, which can be released directly in target tissues in a stable and controlled manner, thereby enhancing their therapeutic index [275]. Many studies have reported that metallic nanoparticles exert strong effects on cellular processes, such as ROS and autophagy [276], and other researchers found that these nanomaterials could inhibit the expression of HIF-1 in cancer cells, leading to cytotoxicity, preventing tumor progression [277,278,279,280].

However, despite the excellent anticancer proprieties reported, some studies showed that these nanomaterials could also repress sirtuin expression and induce oxidative stress, autophagy, and HIF-1 pathways, leading to pro-inflammatory responses and severe toxicity in the cardiovascular, respiratory, and immune systems [281,282,283,284,285,286]. Niska et al. reported that the exposure of human fetal osteoblast cells to TiO_2_-NPs reduced the SIRT3 protein levels, accompanied by mitochondrial ROS and lipid peroxidation [287]. Hence, the decreased expression of SIRT3 after TiO_2_NPs exposure may underlie the nanotoxic effects of TiO_2_-NPs on human osteoblasts.

Cobalt nanoparticles (CO-NPs) induce the HIF pathway by depleting intracellular ascorbate, leading to HIF pathway activation in the lungs and immune system. This study highlights the possible role of HIF hyperactivation in the high failure rates of metal-on-metal hip implants [282]. Hence, the use of nanomedicines for sirtuin and HIF-1 modulation is still limited by some inconveniences, and the clinical translation of the results obtained so far is still a challenge.

Overall, we hope that this deep discussion on nanotherapy-based approaches could stimulate researchers working in connected fields to further study the role of these promising nanostructures in the modulation of more EMT-related pathways. Indeed, the significant versatility that these nanomedical approaches display in interfering with so many intracellular processes could open novel avenues for cancer treatment.

## Figures and Tables

**Table 1:** Connections between sirtuins and hypoxia-inducible-factors HIF-1α and HIF-2α. The deacetylation sites on HIF-1a are reported for SIRT1 and SIRT2. Moreover, the effects on protein stability and transcriptional activity are reported.

Sirtuins	EMT/Hypoxia Related Targets	Enzymatic Activity	Effects	References
**SIRT1**	HIF-1α	Deacetylation on Lys674	p300-HIF-1α Transcriptional Activity inhibition HIF-1α decreased activity Epithelial-like phenotype maintenance	[240,241,242,243]
	HIF-2α	Deacetylation	HIF-2α Increased Activity Metastastic process inhibition	[244,245]
	Notch		Notch decreased activity	[246,247]
	β-catenin	Deacetylation	Wnt-dependent EMT inhibition	[246,247]
**SIRT2**	HIF-1α	Deacetylation on Lys709	HIF-1α hydroxylation and degradation increase Transcriptional Activity inhibition Decreased Stability	[248,249,250,251]
	NF-kB	Deacetylation	NF-kB related genes suppression	[252]
**SIRT3**	MnSOD/IDH2	Deacetylation	HIF-1α indirect inactivation/hydroxylation/proteasomal degradation Cancer growth, angiogenesis, and metastasis inhibition	[253,254,255]
**SIRT6**	HIF-1α	acH3K9 Deacetylation (Chromatin Regulation)	p300 recruitment inhibition HIF-1α transcriptional activity inhibition (glucose uptake and glycolysis decrease/oxidative phosphorylation increase) HIF-1α stability increase in PTC with cancer progression	[256,257,258,259,260,261]
**SIRT7**	HIF-1α HIF-2α	Deacetylation-Independent Activity	Reduced stability Transcriptional Activity inhibition	[262]

## Data Availability

Data is contained within the article.
